# An Investigation of Dairy Cattle Welfare in Commercial Iranian Farms: Results from Management Practices, Resource-Based Measures, and Farm Records †

**DOI:** 10.3390/ani15203001

**Published:** 2025-10-16

**Authors:** Ali Jafari-Gh., Richard Laven, Fatima Khaloubagheri, Saeid Jafari-Gh., Mohsen Haji Mirrahimi, Mehdi Dehghan Banadaky, Kristina Ruth Mueller, Emilie Vallee

**Affiliations:** 1 School of Veterinary Science, Massey University, Palmerston North 4410, New Zealand; r.laven@massey.ac.nz (R.L.);; 2 Independent Researcher, Palmerston North 4472, New Zealand; 3 Animal Science Department, College of Agriculture and Natural Resources, Karaj Islamic Azad University, Karaj 3149968111, Iran; 4 Faculty of Veterinary Medicine, Karaj Islamic Azad University, Karaj 3149968111, Iran; 5 Animal Science Department, College of Agriculture and Natural Resources, University of Tehran, Karaj 3158777871, Iran

**Keywords:** animal welfare, free-stall, bedded-pack, cow comfort, resource-based

## Abstract

**Simple Summary:**

Currently, there is no routine, systematic assessment of animal welfare on Iranian dairy farms. To address this gap, a comprehensive protocol was designed to evaluate the welfare of dairy cattle on Iranian farms. This paper focuses on assessment of resources and management practices on four critical areas of housing, water and feed availability, cow flow management, and hoof health management as well as the data recorded by the farmers (e.g., incidence of lameness and mastitis). We identified significant welfare concerns across all categories. For example, although all pens were equipped with at least one water trough, 6 out of 43 farms provided <3 cm water trough length per cow. Cow flow management was negatively affected by the width (median of 116 cm in free-stall and 180 cm in bedded-pack farms) and quality of transfer passages, a concern amplified by the fact that most farms milked the cows three times daily resulting in prolonged periods away from the pen. Additionally, even though heat stress negatively affects cattle wellbeing on Iranian farms, a substantial number of farms did not use cooling and ventilation systems. These results underscore substantial disparities in welfare conditions among farms and highlight the limitations of broad, high-level welfare assessments.

**Abstract:**

There is currently no routine systematic assessment of welfare on Iranian dairy farms and no industry-recognised welfare assessment protocols. Therefore, we aimed to design a comprehensive welfare assessment protocol and use it to assess dairy cattle welfare on Iranian dairy farms that could serve as baseline data. Out of the 54 farms on which milking time was measured, 14 had cows spending ≥4 h a day for milking. Additionally, 17/43 farms provided <6 cm of water trough length per cow, and 9/46 farms provided <47 cm of feed trough length per cow, falling short of international guidelines. Hoof trimming was considered a routine procedure with 51/56 farms trimming the hooves ≥2 times a year. The main housing problem in bedded-pack farms was lack of resting areas with 18/29 farms providing less space than the minimum requirement of 5.4 m^2^/cow, while in free-stall farms bedding depth was the principal housing issue with 16/28 providing ≤10 cm of bedding. Finally, only 31/42 farms that provided farm data kept a record of more than half of the parameters that we asked for. Our findings indicate that a high proportion of farms did not provide sufficient resources or implement management practices necessary to meet welfare requirements of dairy cattle on Iranian farms.

## 1. Introduction

The change from pastoral/semi-pastoral systems to confined housing systems in many countries has resulted in significant challenges for the dairy industry [1]. These include multiple animal-welfare related challenges, with particular issues related to lameness, mastitis, lying behaviour, agonistic interactions, feed availability, and metabolic disease [2,3,4]. These challenges, and the perception that housed systems are less natural [5], have resulted in intensive housed systems being the principal focus of most welfare assessment schemes for cattle [6].

Most intensive dairy farms in Iran are located in parts of the country where the climate is arid/semi-arid and not suitable for grazing dairy cattle. In Iran, housed dairy cattle produce the majority of milk (approximately 5 million tons of milk of a total of 8.4 million tons produced in the country; personal communication). Despite this, there is no industry-recognised welfare assessment scheme and no routine systematic assessment of cow welfare on Iranian dairy farms.

In our previous paper [7], we described the development of a welfare scheme designed to create baseline data of the welfare of housed dairy cattle in Iran. In that paper we focused on the animal-based and stockmanship measures and showed that “animal welfare” was not a term with which farmers/farm managers were familiar. We found that some animal-based welfare indicators such as body condition score (BCS) were managed well by the farmers (though for production rather than welfare reasons), while some other indicators such as tail damage were neglected.

Animal-based measures are a crucial part of welfare assessment because they are considered a direct and ‘outcome’-based way to assess animal welfare [8]. However, there are some limitations to animal-based measures. For example, not all welfare issues can be identified on a single visit using animal-based measures [8]. Therefore, farm records can be useful because they can provide a timeline of treatments and health interventions (such as mastitis or lameness), as well as detailing the number of deaths and injuries alongside reproductive performance and productivity.

In addition, some potential welfare issues cannot easily be identified using animal-based measures. For example, unlike hunger which can be inferred using BCS and/or rumen fill with minimal stress to the cow, the presence of thirst cannot be easily evaluated at the animal level, so the risk of thirst is usually determined by measuring water availability [9], which is a resource-based measure. Such measures identify risks rather than outcomes, so they are useful for assessing situations where there is not currently a welfare problem but where there could be in the future. Examples of this include heat or cold stress—measuring shade and shelter can estimate the risk of temperature stresses even when the current ambient temperature is favourable. Assessing resources can also be useful even when animal-based assessment has identified a problem, as such assessment can identify key risk factors and areas for improvement.

Therefore, the welfare protocol that we developed for intensive Iranian dairy farms [7] included resource-based measures and farm records alongside animal and stockperson-based measures. This paper presents a descriptive report of the outcome of our management-, resource- and record-based assessments on 62 intensive dairy cattle farms in Iran. We aim to provide baseline data on management practices and available resources that can potentially influence dairy cattle welfare on Iranian data.

## 2. Materials and Methods

All study procedures were approved by the Animal Ethics Committee of the College of Agricultural and Natural Resources, University of Tehran (protocol code 6765D and 22 June 2022).

### 2.1. Study Population

Data were gathered from 62 intensive dairy farms in arid and semi-arid provinces in Iran from May 2022 to March 2023. The aim was to visit at least 60 dairy farms (based on Whay et al. [10]). Farms were chosen using the snowball sampling method [11]. Detailed description of the sampling method is presented elsewhere [7].

### 2.2. Protocol Development

For the resource-based section of the protocol, papers published in North America were the key sources [12,13,14,15] alongside Welfare Quality [16] and a recent New Zealand study [17]. Two more informal sources were also used [18,19]. A shortlist was created and was tested before the study started [7]. The protocol was further changed and developed during the visits as the research team found an area of interest. As the protocol was still being developed during the farm visits, additional measures were added during the process. That means that not all measurements were made on all 62 farms.

### 2.3. Data Collection Process

Data were gathered by four members of the research team: A.J-Gh. (PhD student), F.K (farmer, agronomist), S.J-Gh. (animal scientist), and M.H.M (senior veterinary medicine student). A.J-Gh. and F.K were present during all assessments while S.J-Gh. and M.H.M were present based on availability. Prior to data collection, all four team members underwent a calibration process to establish a shared understanding of the study procedures. During the visits, A.J-Gh. supervised the data collection activities conducted by the other team members to maintain methodological integrity.

**Figure 1:**
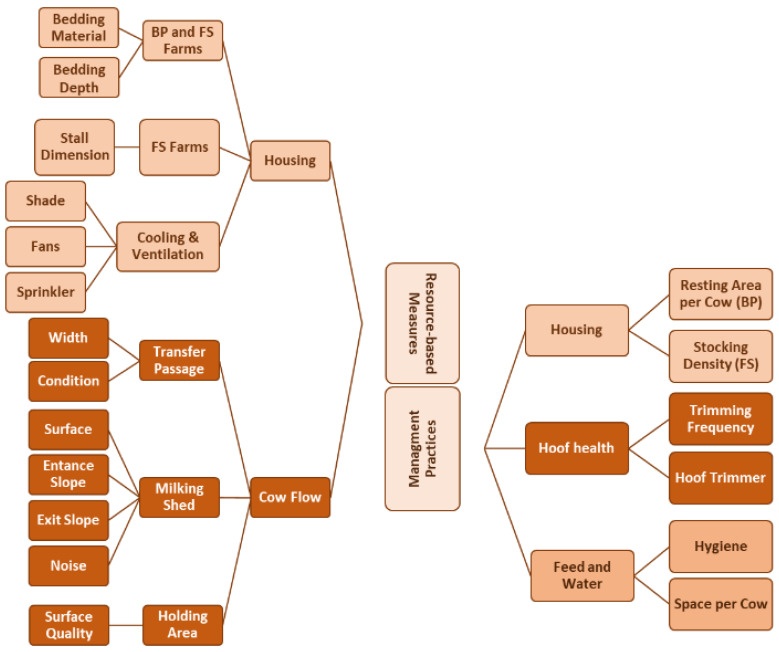
Management practices and resource-based measures assessed during a cross-sectional welfare assessment on 62 Iranian dairy cattle farms. FS stands for free-stall farms, and BP stands for bedded-pack farms.

All data were gathered in a way to minimise stress to the cows. For example, data regarding stall dimension or feed bunk space were gathered only when cows had left the barn for milking, and data regarding the condition of the transfer passages were only gathered when no cows were moving between their pens and the milking parlour. Data were put into five different categories: housing, water and feed availability, cow flow management, hoof health management, and farm records (see Figure 1).

#### 2.3.1. Housing

Measures assessed in this category can be divided into measures that were recorded similarly on bedded-pack (BP) and free-stall (FS) farms (i.e., cooling and ventilation) and measures specific to each system or recorded differently. The latter included resting area per cow (BP only), stocking density and stall dimensions (FS only), and bedding material and depth (both systems).

##### Cooling and Ventilation

Presence or absence of sprinklers/soakers and fans inside the barn, over the troughs, in the parlour holding area, and inside the parlour were recorded separately. Damaged fans or damaged lines of sprinklers/soaker were recorded as “absent”. In addition, the availability of shade (full shade if it covered the whole area, and partial if it did not) over the resting area and troughs were recorded separately.

##### Free-Stall Farms

Measures assessed in FS farms were performed only in pens used to keep early lactation cows (i.e., cows with days in milk of ≤150), and included stocking density (i.e., ratio of cows to undamaged stalls reported as percentage as per [15]), bedding type and depth (at the shallowest part of a stall), presence of a dirty alley (alley covered by a layer of manure ≥2 cm) [14]), and stall dimensions [20], calculated from the average dimensions of five undamaged stalls. The presence/absence of non-usable stalls (damaged stalls that were too narrow, too wide, or had displaced stall bars) was also recorded.

##### Bedded-Pack Farms

Measures included average bedded area per cow of early lactation cows, bedding type, and bedding depth (as the shallowest part with an area of at least one square metre).

#### 2.3.2. Water and Feed Availability

Water trough cleanliness was recorded as per Welfare Quality [16], while feed trough cleanliness was assessed based on a 3-point scoring system: 0—no mould, manure, dirt, rubbish, or sign of contaminants; 1—small amount of old feed residue that could potentially grow mould (e.g., at the corners of the trough wall); 2—clear signs of mould, manure, dirt, trash, or any contaminant.

Both water and feed trough length were measured using a metal rolled tape, with only the usable length measured (e.g., if there was a barrier preventing cows accessing the float, this was not included). NRC [12] recommendations (47 cm/cow) were considered as the threshold for sufficient feed space and Welfare Quality recommendations [16] (6 cm per cow) for water space. Access to warm water during cold seasons, distance to water after milking, and the number of feed deliveries per day were also recorded.

#### 2.3.3. Cow Flow

Resource-based parameters that could potentially affect cow flow were measured and recorded in two distinct areas: milking parlour and transfer passages (pen to parlour).

##### Transfer Passage

Transfer passage width (narrowest part), maximum walking distance to parlour (i.e., longest distance from the parlour exit to the end of a pen), the presence of sharp turns (>90° angles), the presence of narrow gateways (i.e., less width than the transfer passage), and condition of transfer passage were all recorded. Condition was categorised as good (no holes, bumps, or slippery surface), moderate (holes with approximately <10 cm diameter, uneven surface), or poor (major issues that could potentially harm the cows, e.g., holes with approximately >10 cm diameter, and slippery surface).

##### Milking Parlour

**Figure 2:**
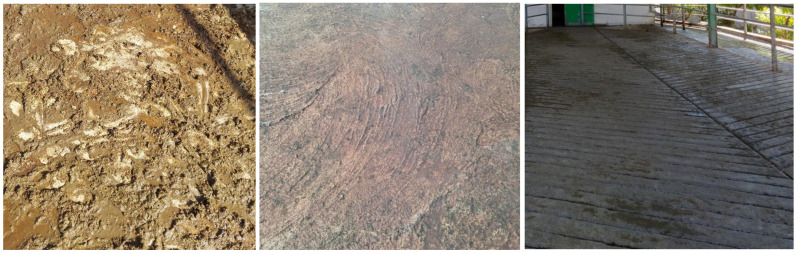
Different surface quality scores in parlour holding area—(**Left**) Score 1: a smooth surface with potential chance of slipping; (**Middle**): Score 2; non-grooved surface, but rough enough to reduce slipping; (**Right**): Score 3; grooved concrete or rubber mats where chances of slipping are low.

Parlour entrance and exit gradient was recorded as suggested by Chesterton [21] and was considered good if the gradient was <10%, average if 10 to 15% gradient, and not acceptable if >15%. Parlour surface (e.g., rubber mat or concrete), sharp turns in the entrance or at the exit (90° angles if the exit transfer passage width was <3 metres), holding area surface quality (Figure 2), absence/presence of cows holding their heads up while waiting in the holding area [17], and maximum return time (time between the first cow leaving the pen and the last cow returning to the pen, [13]) were measured and recorded. Noise levels were classified as 0, 1, or 2 based on the ability of two individuals standing two metres apart to communicate using their normal speaking voice: level 0 if speech was easily heard, level 1 if heard with difficulty, and level 2 if not heard at all (modified version of Sapkota et al. [17]).

#### 2.3.4. Hoof Health Management

Information on hoof trimming frequency (the average number of trimmings per cow per year) and whether the trimmer was a farm worker or an external contractor was collected. Availability of foot bath and a pre-wash bath were recorded, and data on foot bath frequency and the type of solution used collected.

#### 2.3.5. Farm Records

After the welfare assessment, the farmer/farm manager were asked to provide the following farm records: annual (from 21 March 2021 to 21 March 2022) recorded incidence of mastitis, lameness, culling, calf mortality, stillbirth, and current data on herd average 100-day in-calf rate, days open (DO), milk fat and protein yield, average milk production per cow (in litres/day), somatic cell count (SCC), mean lactation length, and current mean days in milk (DIM). All data were recorded as herd average.

### 2.4. Statistical Analysis

All data were recorded on recording sheets before being transferred to Microsoft Excel. Data were double-checked to make sure that there were no transfer errors or misrecordings. Descriptive statistics (i.e., mean, 95% confidence intervals for mean, median, interquartile range, minimum, and maximum) were calculated and boxplots drawn using SAS version 9.4 (SAS Institute, Cary, NZ, USA). Bar charts and descriptive graphs were drawn using Microsoft Excel. DATAtab was used for visualisation purposes [22].

## 3. Results

Overall, 63 farms were visited in arid and semi-arid regions in Iran: Tehran (*n* = 37), Alborz (*n* = 15), Isfahan (*n* = 3), Qazvin (*n* = 4), and Qom (*n* = 4). Data from one farm in Tehran were lost. The remaining farms were put into three size categories based on tertiles (21 farms per category): small (<180 lactating cows), medium (181 to 899 lactating cows), and large (>900 lactating cows). The mean (95% confidence interval; CI) number of cows per pen in small, medium, and large farms were 48 (34 to 63), 89 (74 to 105), and 184 (157 to 211), respectively.

Across the 62 farms, 34 (55%) kept their fresh (approximate DIM ≤ 21) and early lactation cows (DIM approximately 22 to 150) in BP, while 25 (40%) farms kept them in FS, and in 3 (5%) farms some fresh or early lactation cows were kept in FS and some of them in BP yards (mixed system; MIX). Of the 62 farms, 47% (17 FS, 10 BP, and 2 MIX) kept their fresh cows in a separate pen and then transferred them to a pen where early lactation cows were kept, while another 43% (7 FS, 19 BP, and 1 MIX) kept fresh and early lactation cows in the same pen, and 10% (1 FS and 5 BP) kept their cows from across lactation in the same pen.

As the protocol was still being developed during the farm visits, measures that were not initially included were added to the protocol as the research team realised an area of interest. Thus, not all measurements were undertaken on 62 farms.

### 3.1. Housing

#### 3.1.1. Cooling and Ventilation

**Figure 3:**
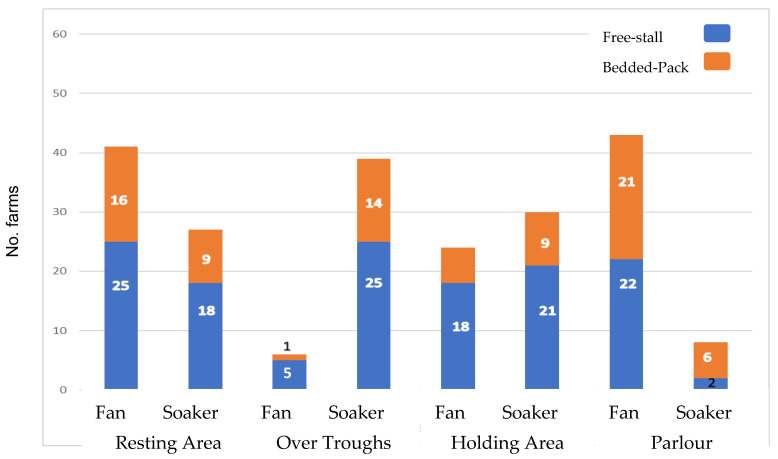
Number of free-stall (blue) and bedded-pack (orange) farms that used fans and sprinkler/soaker at different locations. Numbers inside the bars shows the number of farms in each category (free-stall/bedded-pack), and number on the left axis shows total number of farms that used this equipment.

These measures were recorded on all 62 farms. The use of fans and sprinklers/soakers was observed on 43/62 (69%) and 39/62 (63%) farms, respectively, with 6 (10%) farms not using fans or sprinklers (1 FS and 5 BP). Fans were mostly used over the resting area (41 farms) and inside the parlour (43 farms), while sprinkler/soakers were mostly used over the feed troughs (39 farms), and in the holding area (32 farms) (summarised in Figure 3).

In 55% of farms (21 FS, 12 BP, and 1 MIX), the feed troughs were completely shaded, while in 66% (22 FS, 18 BP, and 1 MIX) the resting area was completely shaded. Partial provision of shade above the feed troughs was observed in 37% of farms (4 FS, 17 BP, and 2 MIX) and above the resting area in 32% of farms (3 FS, 15 BP, and 2 MIX). Five farms provided no shade above troughs and one no shade above the resting area (all BP farms).

#### 3.1.2. Free-Stall Farms

Data related to stall dimensions and stocking density in the 28 farms which used FS (FS and MIX farms) are shown in Table 1. Median (interquartile range; IQR) stocking density was 95% (85 to 121%), i.e., 95 cows per 100 undamaged stalls. Damaged stalls were observed in 12/28 (43%) farms. Bedding materials used included dried manure (14/28), sand (12/28), and wood chip (2/28). The feed alley was dirty in 13/28 (46%) farms. All farms had concrete feed alleys except one farm which had installed rubber mats.

#### 3.1.3. Bedded-Pack Farms

**Figure 4:**
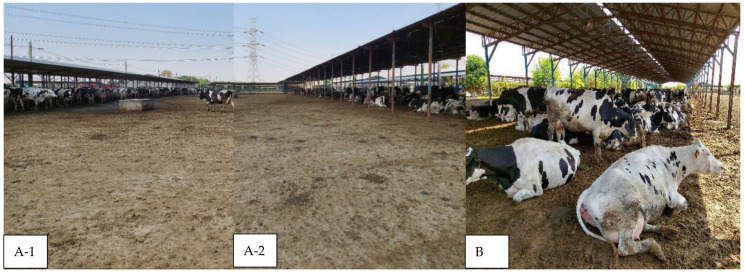
An example of a bedded-pack farm in Iran. The farm can be composed of multiple pens depending on farm size. Each pen has a bedded section (**B**) where cows can rest and a big concrete walking area with access to feed and water troughs (**A-1**,**A-2**). The troughs are mostly the surrounding sides (in this example, there is a water trough in the walking area in (**A-1**)) and are mostly accessible from outside the pen by a wide track where tractors and feeders can move. Dried manure solids are the main source of bedding, and bedding depth varies from thin (as in the photo (**B**)) to very thick.

Overall, 37 farms (34 BP and 3 MIX) kept fresh and early lactation cows on bedded packs. Figure 4 shows a typical pen on an Iranian BP farm. Dried manure was the only source of bedding on all farms for all lactation groups other than fresh (approx. DIM ≤ 21) cows. On 5/12 farms, fresh cows were kept on straw-topped manure, while on 2/12 farms they were kept on a mix of wood chips and dried manure solids, and in 5/12 farms fresh cows were kept on dried manure solids.

The resting area available to fresh (approx. DIM ≤ 21) and early lactation cows (approx. DIM 22–150) was recorded on 9 and 25 farms, respectively. Median (interquartile range; IQR) resting area per fresh cow was 808 cm^2^ (571 to 1042 cm^2^) and for early lactation cows was 463 cm^2^ (300 to 485 cm^2^). Table 1 summarises data regarding bedding depth and resting area per head in BP farms.

### 3.2. Water and Feed Availability

Feed and water trough hygiene were recorded on 62 and 61 farms, respectively, while feed trough length per head for fresh and early lactation cows was recorded on 17/29 and 46/62 farms, respectively. Additionally, water trough length per fresh and early lactation cow was recorded in 16/29 and 43/62 farms, respectively.

**Figure 5:**
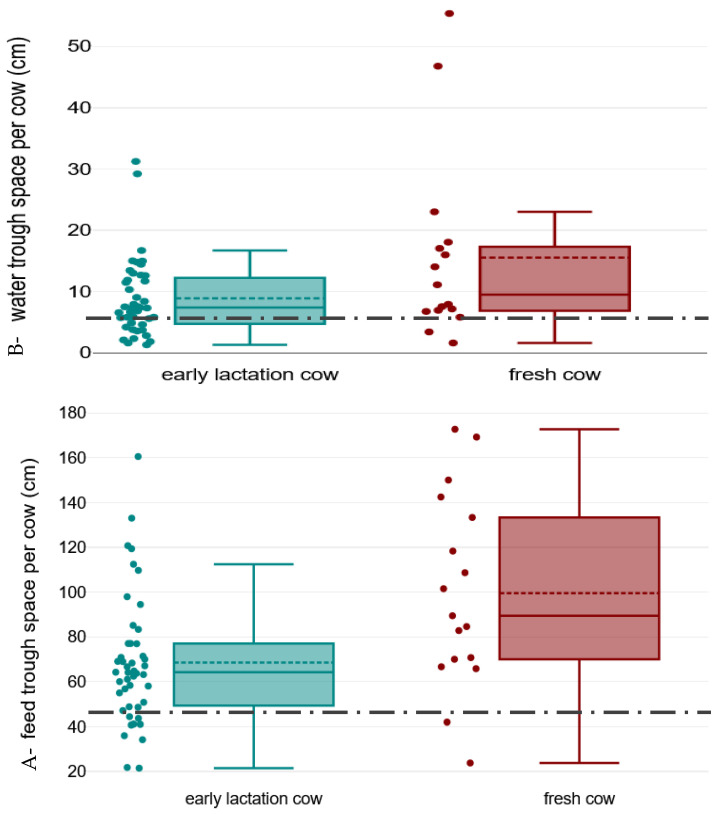
Distribution of available feed (**A**) and (**B**) water trough space per head of fresh (i.e., days in milk 1 to 21) and early lactation cows (i.e., days in milk 22 to 150) and the minimum space requirements (shown by black dotted line) in a cross-sectional study of 62 Iranian dairy farms. The solid line in box shows median, dotted line shows mean, lower end of the box shows first quartile, top end of the box shows the third quartile, and the whiskers show 95% confidence intervals. Dots show the farms.

Figure 5 illustrates the distribution of the available feed (A) and water (B) trough length per cow on the study farms, and Table 1 provides the latter data as well as data regarding distance to water after parlour exit. All farms had at least one water trough in each pen, but 10% of farms (2 FS, 2 BP, and 2 MIX) also provided access to water right after the parlour exit. There were 17 farms (28%; 7 FS and 10 BP) who did not provide the minimum space per head required for water, with 10% of farms (1 FS and 5 BP) providing less than 3 cm water trough length per head. For feed, 3/9 farms which provided insufficient space were FS and 6/9 were BP.

In 26% of farms (6 FS, 9 BP, and 1 MIX), cows accessed clean water in clean water troughs (score 0 of 2), while 36% of farms (14 FS and 8 BP) provided dirty water in clean troughs (score 1), and 38% of farms (5 FS, 16 BP, and 2 MIX) provided unclean water in dirty troughs (score 2 of 2;). In one small BP farm that had 26 lactating cows in one pen, the water trough was empty at the time of visit and could not be scored. Empty water troughs were observed on pens with cows inside them (these pens were outside our sample population) on 2 FS and 5 BP. Feed troughs were clean (score 0) in 35% of farms (13 FS, 7 BP, and 2 MIX), unclean (score 1) in 50% (11 FS, 19 BP, and 1 MIX), and dirty in 15% of farms (1 FS and 8 BP). Feed delivery frequency was not recorded on the first 14 farms visited. Feed was delivered twice a day on 42% farms (1 FS, 14 BP, 1 MIX), three times per day on 48% (10 FS, 12 BP, 1 MIX), and four times a day on 10% farms (4 FS and 1 BP).

### 3.3. Cow Flow

#### 3.3.1. Transfer Passage

**Figure 6:**
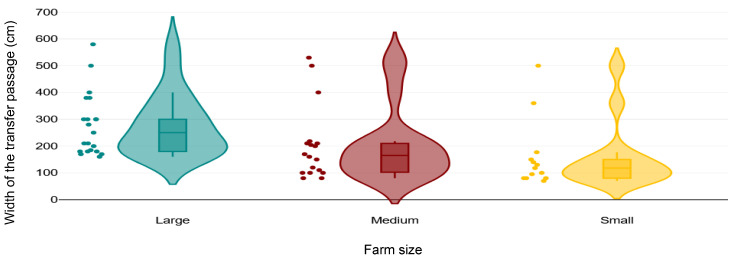
Distribution of different transfer passage widths on small (<180 lactating cows), medium (181 to 899 lactating cows), and large (>900 lactating cows) farms in a cross-sectional study of 62 Iranian dairy farms. The solid line in box shows median, lower end of the box shows first quartile, top end of the box shows the third quartile, and the whiskers show 95% confidence intervals. Dots show the farms. Colour areas show the distribution of the data.

Across all 62 farms, 8 (all small and BP) kept cows in pens next to the parlour, so they had no transfer passages. Transfer passage width and condition were recorded on 52 and 48 farms, respectively. Sharp turns, narrow gateways, and maximum walking distance were recorded in 45, 42, and 38 farms, respectively. Overall median (IQR) transfer passage width was 180 cm (120 to 300 cm; Table 1; Figure 6). For small, medium, and large farms, median (IQR) widths were 118 cm (80 to 150 cm), 185 cm (105 to 214 cm), and 265 cm (180 to 340 cm), respectively. Transfer passage condition was good in 52% farms (16 FS and 9 BP), moderate in 17% (5 FS and 2 BP), and poor in 31% (7 FS and 8 BP). Sharp turns in the transfer passages were observed in 53% of farms (16 FS and 8 BP), whereas narrow gateways were observed in 14% of farms (1 FS, 4 BP, 1 MIX). The median (IQR) maximum walking distance from a pen to the parlour (one-way only) was 160 m (120 to 230 m; *n* = 38).

#### 3.3.2. Milking Parlour

Number of milkings per day and observing cows with “heads up” in the holding area were recorded in 58 farms, while return time was recorded on 54. Use of rubber mats inside the parlour and in the holding area was recorded on 62 farms, but the quality of surface in holding area was recorded on 56 farms. Entrance and exit slopes were recorded on 59 farms and existence of sharp turns after parlour exit on 48 farms. Noise level was recorded on 61 farms.

**Figure 7:**
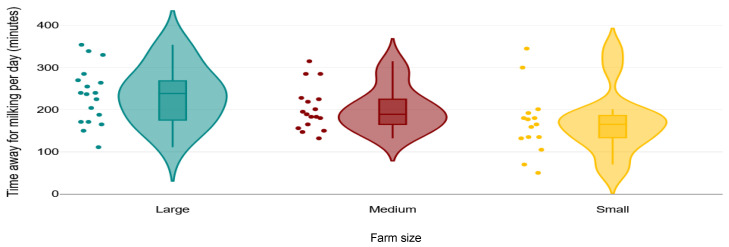
Comparing the daily time away for milking in small (<180 lactating cows), medium (181 to 899 lactating cows), and large (>900 lactating cows) farms in a cross-sectional study of 62 Iranian dairy farms. The solid line in box shows median, lower end of the box shows first quartile, top end of the box shows the third quartile, and the whiskers show 95% confidence intervals. Dots show the farms. Colour areas show the distribution of data.

Cows were milked twice a day in 4 BP farms, three times a day in 52 farms (24 FS, 25 BP, 3 MIX), and four times a day in one FS farm (*n* = 57). Median (IQR) return time from each milking was 61 (50 to 80) minutes (Table 1; Figure 7). Cows were observed with “heads up” while waiting in the holding areas in 53% of farms (12 FS, 16 BP, 3 MIX). The holding area and parlour surface were covered (either fully or partially) with rubber mats in 26% (10 FS and 6 BP) and 53% of farms (19 FS, 13 BP, 1 MIX), respectively. The holding area surface was slippery in 1 FS farm. Median (IQR) gradients for parlour entrance and exit were 5% (3 to 8%) and 5% (3 to 10%), respectively. Two farms had an exit gradient of >25%. Overall, 41/62 of farms (66%) had an entrance and exit gradient of ≤10%. Sharp turns at parlour exit were observed on 65% of farms. Noise levels were 0 in 54%, 1 in 33%, and 2 in 13% of farms.

#### 3.3.3. Hoof Health Management

We recorded the number of hoof trimmings per year on 56 farms (excluding the first four farms visited and two farms who could not provide the information). We recorded who did the trimming on 50 farms. Availability and frequency of foot bath use and product used were recorded on 53 farms, with availability of a pre-wash bath on 41 farms. Functional hoof trimming was performed on all but one farm (55/56). Twenty-one percent of farms (9 FS and 3 BP) trimmed >2 times/year, 70% twice a year (10 FS and 28 BP, 1 MIX), and 7% trimmed <2 times a year (2 FS, 1 BP, 1 MIX). Forty-two percent of farms (9 FS and 11 BP) used trained staff for hoof trimming, while 58% (10 FS, 17 BP, 2 MIX) hired contract hoof trimmers (*n* = 50). Additionally, 57% (16 FS, 12 BP, 2 MIX) of farms had foot baths, but only 10% (2 FS and 2 BP) of the 41 farms that were assessed had pre-wash baths before the foot bath. Two of the 30 farms with foot baths did not use them (both BP), Of the remaining 28 farms with a foot bath, 19 farms used the foot bath ≥3 times a week, 4 weekly, and 5 as needed. Formaldehyde was the main solution used in the foot baths (27/28).

### 3.4. Farm Records

All farms were asked to provide their recorded incidence of mastitis, incidence of lameness, culling rate, calf mortality rate, days open (all based on yearly data), and their latest record of average milk yield per cow, milk fat percentage, milk protein percentage, milk somatic cell count (SCC), and herd average days in milk (DIM). Overall, 42 farms (16 FS, 23 BP, 3 MIX) provided some data; however, only 31 farms (13 FS, 15 BP, 3 MIX) provided >50% of the data we asked for. The main reasons for not providing data were not having a reliable record (*n* = 28/62; especially for the incidence of lameness and mastitis), not being able to access the data (e.g., the person responsible for farm records was not working on that day, or they did not keep the records; *n* = 15/62), and being too busy; *n* = 11/62). Median (IQR) farm recorded incidence of lameness, mastitis, and rate of calf mortality were 8.3% (6.1 to 18.0%), 14.6% (5.4 to 23.6%), and 6.1% (3.5 to 9.6%), respectively. Table 2 provides a full summary of farm records.

## 4. Discussion

Resource-based measures and management parameters are crucial parts of a welfare assessment protocol and are important to improve welfare, reduce risks, and make interventions [4,9]. This is the first study of welfare-related resources, management practices, and records on Iranian dairy farms and thus provides a baseline for monitoring improvements and changes.

The focus of our assessment of resources and management practices was on the critical areas of housing, water and feed availability, cow flow management, and hoof health management. In all areas, we identified issues where a high proportion of farms were not providing sufficient good quality resources. Even though heat stress negatively affects cattle performance and wellbeing on Iranian farms [23,24], a substantial number of farms did not use cooling and ventilation systems (e.g., 48% of farms had no fans over holding areas and 37% had no sprinklers). This was exacerbated by a lack of shade over feed and water troughs (45% of farms no/partial shade), over-crowded holding areas (53% of farms had cows with heads-up which is a welfare measures used frequently on New Zealand farms), and a lack of resting areas on BP farms (75% with <7 m^2^ and 28% with ≤3 m^2^). This shows a lack of understanding of the importance of heat abatement for dairy cows, especially in holding pens which are the areas where cows are most prone to heat stress [25]. Furthermore, cows with insufficient shade will show more aggressive behaviour and spend more time around water troughs [26], so in such cases access to water is key to cow welfare. Our data suggest that cows on high-density farms may have less access to water (e.g., 4/7 farms with ≤3 m^2^ shaded resting space had ≤2.5 cm/head water trough space). On such farms, cows are likely to be doubly compromised by the lack of shade.

Our data highlight that lack of heat stress abatement is an issue on many Iranian dairy farms, demonstrating the need to move from the high level, perhaps simplistic assessment, used in this study to a deeper level. For example, we did not differentiate between sprinklers, foggers, and soakers. Some farms used sprinklers and foggers over the trough and in holding areas even though it is recommended that soakers be used on such areas [25]. We need to actively measure the heat abatement ability on such farms. Similarly, we only recorded the presence/absence of fans rather than their flow rate and their cooling effect.

For free-stall housing, the main problems were identified around bedding depth and stall size. Bedding depth was low on many farms, with ≤10 cm on 57% farms and ≤5 cm on 39% (*n* = 28). However, it is possible that these low bedding depths could be mitigated by good stall size [27]. The median size for all stall parts on Iranian farms meets the international requirements, except for stall length in single-row stalls and brisket length (in both single and double rows). Despite this, data suggest that there were a substantial number of farms at the lower end of the spectrum that did not meet these requirements. For example, when comparing to NFA [28], the Norwegian standard for dairy cows weighing 550 to 650 kg live body weight, 5/25 farms did not meet the stall width requirements while 6/9 single row stalls did not meet the stall length requirements.

Another issue was dirty feed alleys in 46% (*n* = 28) farms in this study. This issue is clearly linked to the high prevalence of dirty legs in the same farms [7], and demonstrates that further research is needed to better understand what is driving the poor cleanliness on many Iranian free stall farms, such as whether it is associated with a method of cleaning.

During the study, it became clear that multiple farmers thought that simply installing free-stalls could improve cow comfort and productivity without considering other changes that are needed, such as the need to install fans or sprinkler/soakers and the continued maintenance costs associated with bedding. One recently converted farm had a zero percent stall usage index, and another one had the highest prevalence of lame cows (86%) across the whole study. Clear signs of heat stress (panting and high respiratory rates) were observed on both farms.

The key housing issue on BP farms was space per cow. Across the 37 farms, available resting area ranged from <1.5 to >15 m^2^, with only 38% (*n* = 29) farms having more than the minimum space requirement of 5.4 m^2^ per cow [29]. We need more data on the factors driving the low space per cow on Iranian dairy farms and the effect of this on cow behaviour as well as productivity, especially for the latter, as current published evidence of the effect is equivocal [30,31,32].

For water and feed availability, trough space was insufficient on too many farms (Figure 5). Twelve out of forty-three (28%) farms did not provide the recommended 6 cm per cow water trough length [16], and four (9%) farms failed to provide half of the recommended space. This was exacerbated by 8/62 (13%) farms having at least one pen with no water at the time of the visit and cows not having access to clean water in more than one third of farms, which can impair cows’ behaviour [33]. Research shows that this lack of access to water in dairy cattle farms is a global problem [34,35,36], but for our study farms it is of particular importance as all the farms were located in arid and semi-arid regions (so at high risk of heat stress) [37], and all cows were fed TMRs with high dry matter contents.

There were similar, though perhaps less severe issues with feed troughs. Space per head was less than recommended (i.e., 47 cm per cow) in 20% of farms (*n* = 46). Additionally, troughs were dirty in 9/62 (15%) farms, 2 of which had <47 cm feed trough length per cow. This lack of attention to trough management contrasts with our findings regarding nutrition management on these farms [7] and the low number of cows with low body condition score. Further research is needed to investigate the welfare and productivity impact of poor trough management on welfare in Iranian dairy farms.

Transfer passage width ranged from 70 cm to 580 cm, and it was <130 cm in 14/52 (27%) farms. However, the width requirements vary depending on the number of cows per herd. We suggest a minimum transfer passage width of 130 cm on any farm to allow two cows to use the transfer passage at the same time and avoid congestion caused by lame or distracted cows, which might in turn encourage the stockperson to use force to move the cows. However, larger herds probably need larger passages. A minimum of 500 cm [38] might be useful for large farms (>900 lactating cows in this study) where herds of >120 cows are regularly transferred through the lanes, while for medium farms (181 to 899 lactating cows in this study) a minimum of 244 cm would be useful [39]. Using these standards, 2/21 large farms (10%), 4/20 medium farms (20%), and 5/12 small farms (42%) met the requirements (Figure 6). In addition, cows were deprived of a good transfer passage (i.e., score 0 in a 0 to 2 scoring system; no hazards) in 48% of farms, while there were potential hazards (i.e., scores 1 and 2 in a 0 to 2 scoring system; existence of big holes or slippery surface) in the transfer passages in 31% of farms (*n* = 48). Few farms (6/42) had narrow gateways, yet 24/45 farms (53%) had sharp turns in the transfer lanes. These findings show that there is potential disruption in cow flow during milking times, which may encourage the stockperson to use force to move the cows along [7]. This lack of maintenance of the transfer passages and poor passage design might also contribute to the high prevalence of lameness on Iranian farms [40].

Multiple milkings per day meant that cows spent a considerable proportion of their day away from their pen for milking, with 9/54 (17%) spending 4 to 5 h/day and 5/54 (9%) spending 5 to 6 h (Figure 7). In addition, cows stood on concrete in 74% holding areas with only 26% farms using rubber mats (*n* = 62). This long milking time combined with lack of floor mats on the holding area might lead to increased odds of lameness on the farms [41] and a change in behaviour in cows [42].

The parlour entrance was steep (i.e., >10%) in 12.5% and too steep (i.e., ≥15%) in 8% of farms, while the exit was steep in 17.9% and too steep in 3.4% of farms. We need further data on the impact of these structural issues particularly in combination with the poor handling as we previously reported [7]; this can result in increased prevalence of lameness and impaired cow welfare [43,44,45].

Consistent with other data from Iran [46], hoof trimming was a routine procedure on most of our farms. A recent large-scale study in the UK showed that 82% of the farms performed preventive trimming with 46% solely using a contractor and 32% solely using farm staff [47]. This is comparable to our study as 58% farms used a contract hoof trimmer and 42% used farm staff (*n* = 50). A range of trimming frequencies was observed from none to three times per year, but two times was the most common frequency (39/56). On the one farm which did not trim, this was because the farmer did not “believe in trimming” despite having a lameness prevalence of 86%.

However, although trimming was common, we have very little evidence of how effective it was. One particular issue is that most people trimming hooves are trained by other hoof trimmers, with no official organisation or educational provider delivering trimming courses. The high median prevalence of lameness on our study farms (33%) [7] despite the routine use of foot trimming strongly suggests that research is urgently needed on the quality of hoof trimming on Iranian dairy farms and its impact on lameness risk and whether the prevalence of lameness on Iranian farms is associated with trimming frequency on the farms.

In addition, most farms (57%) had a foot bath and used it several times a week. This is comparable to dairy farms in Minnesota where 68% of farms used foot baths [41]. That more than half the farms used a foot bath might suggest a potentially high risk of digital dermatitis infection on the farms, which is in contrast to previous reports (reported prevalence of 12% on four commercial farms) [48]. In addition, 27/28 farms reported using formaldehyde, which is classified as a probable human carcinogen [49].

Farm records were the final area which we evaluated on our study farms. The main conclusion is that, on most of our farms, recordkeeping was less than optimal. This is despite on-farm computer-based recording being available in Iran since the 1980s [50], and Iranian producers of farm management programmes regularly holding free courses for farmers and farm staff on how to use their software.

Even where there were records, the disease-specific records lacked reliability with the recorded incidence of lameness (8.3%) not matching the prevalence of lameness identified as part of the study (33%) on these farms [7], and farm records regarding the incidence of mastitis (24%) contrast with a previous report by Hashemi et al. [51] identifying a 2% prevalence of clinical and 44% prevalence of subclinical mastitis on dairy farms in southern Iran. However, data regarding calf mortality or culling might be more reliable as it seems easier to record the number of dead calves or culled cows compared to the number of cows dealing with lameness or mastitis. The most reliable set of data are assumably those regarding milk production (i.e., milk yield, milk fat and protein percentage, and SCC) as these data are provided by dairy companies based on tests performed in their laboratories.

## 5. Conclusions

This study assessed resource-based welfare measures in 62 Iranian intensive dairy farms located in arid and semi-arid areas. The two main farming systems used were free-stall farms (FS; with or without a walking yard) and bedded-pack farms (BP). Overall, FS farms used more fans and sprinklers in the resting area, over the troughs, and in parlour holding areas. Overcrowding was more observed in BP farms as five out of the six farms that provided <3 cm of water trough space/head were BP, and a clear lack of available resting space was observed in BP farms as well. Our results show that some farmers might underestimate the importance of providing shade for the cows as more farms provided fans and sprinklers than full shade over the troughs and resting area. Additionally, cows in almost half of the studied farms were deprived of a good walking surface in the transfer passages. The resource-based measures used in this study can be used to assess the nutrition and physical environment domains of dairy cattle welfare (based on the 5-domain model) and can be a complement to the animal-based and stockperson-based measures (subjective measures that can cover health and behavioural interaction domains) in a thorough welfare assessment protocol.

## Figures and Tables

**Table 1:** Distribution of different resource-based welfare measures in a cross-sectional study of 62 Iranian dairy farms.

Measure	Unit	Mean	95% CI for Mean		First Quartile	Median	Third Quartile	Minimum	Maximum	*n*
			Lower	Upper						
**Feed and water availability**										
Feed space/head early lactation cow ^1^	cm	68.5	60.1	77.0	48.8	64.2	77.1	21.5	160.5	46
Feed space/head fresh cow ^2^	cm	99.6	77.4	121.7	70.0	89.5	133.3	23.8	172.7	17
Water space/head early lactation cow	cm	8.87	6.91	10.8	4.60	7.37	12.6	1.30	31.2	43
Water space/head fresh cow	cm	15.5	7.47	23.6	6.83	9.51	17.6	1.60	55.4	16
Distance to water after parlour	m	77.7	56.6	98.9	30.0	50.0	100	0	380	59
**Housing in bedded-pack farms**										
Bedding depth in bedded-pack farms	cm	20.3	14.7	25.9	12.5	15.0	30.0	2.0	80.0	32
Resting area/head early lactating cow	cm^2^	575	420	730	300	463	485	125	1558	25
Resting area/head fresh cow	cm^2^	784	505	1063	571	808	1042	452	1586	9
**Housing in free-stall farms**										
Bedding depth in free-stall farms	cm	12.6	8.52	16.6	5.0	10.0	17.5	2.0	50.0	28
Stocking density in free-stall farms ^3^	%	98.7	86.6	110.7	85.3	95.2	121.0	33.3	139.5	21
Length—single-row stalls	cm	230	214	246	215	228	240	210	275	9
Length—double-row stalls	cm	247	238	256	230	255	260	205	273	21
Width	cm	116	114	118	115	116	120	105	125	24
Brisket height	cm	20.4	3.47	37.4	10.0	15.0	25.0	8.0	60.0	7
Low loop height	cm	33.3	29.2	37.3	28.0	32.0	40.0	18.0	60.0	23
Loop diameter	cm	77.0	74.4	79.6	74.0	77.0	80.0	64.0	92.0	23
Neck rail height	cm	124	121	127	116	125	130	115	135	23
Curb to brisket	cm	220	201	239	205	215	225	205	265	7
Rear curb width	cm	15.2	12.3	18.0	10.0	15.0	20.0	8.0	33.0	23
Rear curb to neck rail	cm	163	155	171	160	165	170	115	195	23
Rear curb to stall divider	cm	34.1	27.6	40.5	25.0	30.0	40.0	13.0	75.0	23
**Cow flow management**										
Returning time per milking ^4^	min	66.2	59.9	72.4	50.0	61.0	80.0	25.0	118	54
Milking shed entrance slope	%	6.0	4.7	7.3	3.0	5.0	8.0	0	21.0	59
Milking shed exit slope	%	6.8	5.3	8.3	3.0	5.0	10.0	0	25.0	59
Transfer passage width	cm	225	187	262	120	180	300	70	580	52
Maximum walking distance	m	193	153	232	120	160	230	50	560	38

^1^ Cows with days in milk of 22 to <150; ^2^ cows with days in milk of 1 to 21; ^3^ number of cows per 100 stalls; ^4^ time interval between when the first cow leaves the pen and when the last cow returns to the pen.

**Table 2:** Distribution of farm recorded data regarding welfare issues and performance indicators on 62 Iranian dairy farms.

Farm Recorded Data ^1^	Unit	Mean	95% CI for Mean		First Quartile	Median	Third Quartile	Minimum	Maximum	N ^2^
			Lower	Upper						
Mastitis ^3^	%	18.2	10.9	25.6	5.4	14.6	23.6	1.40	77.0	25
Lameness ^3^	%	12.8	9.11	16.4	6.05	8.25	18.0	1.70	38.7	32
Culling	%	27.5	21.4	33.5	18.0	25.0	33.1	5.20	65.3	28
Calf Mortality	%	7.31	5.21	9.40	3.47	6.10	9.57	0	28.3	31
Days Open	days	138.7	126.4	151.1	121.0	133.0	150.0	103.0	238.0	22
Average Milk Yield	Litre/day/cow	35.4	34.1	36.6	33.2	36.0	38.0	26.0	43.6	42
Milk Fat	%	3.51	3.42	3.61	3.40	3.50	3.70	2.70	4.00	35
Milk Protein	%	3.13	3.07	3.19	3.02	3.09	3.20	2.90	3.50	26
Milk SCC × 1000	cells/mL of milk	244.8	184.5	305.0	185.0	214.5	259.0	63.0	800.0	24
Days in Milk	days	178	170	186	158	179	190	148	230	30

^1^ Data are for the entire herd and not the sample population; ^2^ N represents the number of farms who provided the data; ^3^ these measures were recorded as incidence (i.e., total number of mastitis cases per annum).

## Data Availability

Data can be accessed through the first author based upon request.
